# DeepSnap: From Three-Dimensional Molecular Images to Quantitative Structure–Activity Predictions

**DOI:** 10.3390/ijms27114965

**Published:** 2026-05-30

**Authors:** Yoshihiro Uesawa

**Affiliations:** Department of Medical Molecular Informatics, Meiji Pharmaceutical University, 2-522-1 Noshio, Kiyose 204-8588, Tokyo, Japan; uesawa@my-pharm.ac.jp

**Keywords:** DeepSnap, molecular image, quantitative structure–activity relationship, convolutional neural network, transfer learning, ensemble learning, Tox21, ADME prediction, molecular informatics, explainable artificial intelligence

## Abstract

Quantitative structure–activity relationship (QSAR) modeling has conventionally relied on expert-designed molecular descriptors to encode chemical structures. DeepSnap is a descriptor-free QSAR approach that converts prepared three-dimensional molecular conformers into image representations and feeds them directly into convolutional neural networks for activity prediction. This focused narrative review traces DeepSnap from its introduction in 2018 to its current state and places it within the broader landscape of descriptor-based QSAR, topology-based and 3D-aware graph neural networks, and related image-based or semi-image-based molecular representation approaches. Previous studies applied DeepSnap to Tox21 nuclear receptor and molecular initiating event endpoints, rat hepatic clearance, blood–brain barrier penetration, acute oral toxicity, and cosmetics–pharmaceutical compound classification. Across the DeepSnap series, image-based and descriptor-based predictions have provided complementary information, particularly in ensemble or consensus models. However, high or near-ceiling ROC–AUC values reported for selected endpoints should not be interpreted as indicating deterministic or universally generalizable predictions; rather, they should be considered in the context of endpoint-specific model development, image-rendering parameter optimization, possible class imbalance, split dependence, limited matched external replication, and incomplete benchmarking against modern molecular representation models. Limitations include a dependence on nonphysical rendering parameters, single- or representative-conformer input, incomplete matched benchmarking against 2D and 3D molecular representation models, and an interpretability gap addressed in part by CAM-family visualization in the AI-based Substance Hazard Integrated Prediction System (AI-SHIPS) and S-COPHY (a model developed by Shiseido for cosmetics–pharmaceutical compound classification). Future directions include standardized image-generation protocols, conformer-ensemble extensions, systematic interpretability analysis, matched benchmarking, and potential integration with graph-based and 3D-aware molecular learning approaches.

## 1. Introduction

Quantitative structure–activity relationship (QSAR) modeling has long been a cornerstone of computational chemistry—a topic of direct relevance to the molecular informatics community—enabling the prediction of biological activity, toxicity, and pharmacokinetic properties from molecular structure alone. In drug discovery and chemical safety assessment, QSAR provides a means to prioritize compounds for experimental testing, thereby reducing cost and animal use [1]. The Tox21 initiative, a multiagency collaboration involving the U.S. Environmental Protection Agency, the National Institutes of Health, the National Center for Advancing Translational Sciences, and the Food and Drug Administration, accelerated this field by generating high-throughput screening data for thousands of environmental chemicals across dozens of biological targets [1,2,3]. Within the Tox21 framework, molecular initiating events (MIEs)—the initial interactions between chemicals and biological macromolecules—represent the entry points of adverse outcome pathways (AOPs), linking chemical exposure to toxicological effects at the cellular, organ, and organism levels [1,2,4]. Therefore, predicting which MIEs a given compound may trigger is central to modern computational toxicology.

Traditional QSAR approaches rely on expert-designed molecular descriptors to encode structural information [5]. These descriptors range from simple physicochemical properties such as molecular weight and partition coefficient (logP) to more elaborate representations such as topological indices, extended-connectivity fingerprints (ECFP) [6], and three-dimensional (3D) steric parameters such as Taft, Sterimol, and A-Value indices [2]. Although these hand-crafted features have supported decades of productive modeling, they impose an intrinsic limitation: the quality and completeness of the resulting models depend on the choice of descriptors, which in turn depends on domain expertise and prior chemical knowledge. Descriptor selection introduces human bias, and it is unlikely that any finite set of precomputed features can exhaustively represent the diversity of molecular recognition events. The Tox21 Data Challenge 2014, a landmark competition in which teams from 18 countries predicted the activity of compounds across 12 MIE targets, demonstrated the promise and the ceiling of conventional approaches: the overall winning team, DeepTox, employed deep neural networks trained on ECFP fingerprints and computed descriptors [7]; however, black-box limitations and descriptor dependence remained inherent constraints.

The emergence of deep learning in the early 2010s opened new avenues for molecular property prediction by enabling models to learn representations directly from raw input data, potentially bypassing the feature-engineering bottleneck. Three principal strategies for encoding molecular information in deep learning frameworks have gained prominence. First, descriptor-based deep learning extends classical QSAR by feeding precomputed fingerprints or descriptor vectors into deep neural networks, as exemplified by DeepTox [7]. Second, graph neural networks (GNNs) represent molecules as graphs in which atoms are nodes and bonds are edges, enabling message-passing operations to learn atom-level features from molecular topology. The message-passing neural network (MPNN) framework [8] and its directed variant, D-MPNN (Chemprop) [9], established graph-based methods as a leading paradigm, whereas attention-based architectures such as AttentiveFP introduced interpretable atom-level attention maps [10]. These widely used molecular GNNs generally operate on two-dimensional (2D) molecular topology. In parallel, however, 3D-aware GNNs have been developed to incorporate geometric information such as atomic coordinates, interatomic distances, or directional relationships. A representative example is SchNet, which uses continuous-filter convolutional layers to model atomistic systems from molecular geometry [11]. Third, image-based approaches feed visual or image-like representations of molecules into convolutional neural networks (CNN) originally developed for image classification. Toxic Colors predicted Tox21 toxicity endpoints directly from 2D graphic images of compounds [12], Chemception applied an Inception-ResNet CNN to 2D structural drawings [13], and ImageMol employed self-supervised pretraining on 10 million molecular images [14]. A recent systematic review of image-based molecular representation learning for drug development proposed a taxonomy based on computer-vision learning paradigms [15]. Other related approaches include DEEPScreen, which uses 2D structural compound representations for drug–target interaction prediction [16], and MolMap/MolMapNet, which maps descriptors and fingerprints into 2D feature maps for CNN learning [17].

Against this backdrop, the DeepSnap method was introduced in 2018 as a descriptor-free QSAR approach that converts 3D molecular structures into image-based representations [18]. Rather than computing molecular descriptors or constructing a molecular graph, DeepSnap generates a conformer of each compound, renders it as a colored molecular image, and captures multiple 2D snapshot images from systematically varied viewing angles around the x-, y-, and z-axes. These images are then fed directly into a CNN—utilizing transfer learning from an ImageNet-pretrained model [2]—for property prediction. Therefore, the distinction between DeepSnap and other molecular learning approaches is not simply the presence or absence of 3D information. Rather, DeepSnap represents conformer-derived 3D information as multiview molecular images for CNN input, whereas 3D-aware GNNs incorporate geometric information directly into graph-based architectures, and other image-based methods typically use 2D depictions or descriptor-derived feature maps.

Since its introduction, DeepSnap has undergone substantial technical evolution and has been applied to a broad range of biological targets. The method has been extended from a single Tox21 endpoint to 35 nuclear receptor agonist and antagonist models [19] and 59 MIE models spanning the full Tox21 target panel [20]. Applications have expanded beyond toxicological endpoints to include pharmacokinetic parameters such as hepatic clearance [21] and blood–brain barrier permeability [22]. The computational pipeline has migrated from a Deep Learning GPU Training System (DIGITS)/Caffe/Jmol system to a TensorFlow/Keras/PyMOL framework [20], and an ensemble strategy combining image-based and descriptor-based predictions has demonstrated consistent improvements over either approach alone [21,22]. A subsequent study within the broader DeepSnap research line adapted the method for cosmetics safety assessment and reported gradient-weighted class activation mapping (Grad-CAM) visualizations of molecular features in the S-COPHY model [23].

This review provides a comprehensive account of the DeepSnap method from its conceptual origin to its current state. Section 2 describes the rationale and the first publication. Section 3 details the core rendering and learning pipeline, including the systematic optimization of imaging parameters and the transition between computational platforms. Section 4 and Section 5 survey applications to toxicological targets and absorption, distribution, metabolism, and excretion (ADME) parameters, respectively. Section 6 traces the technical evolution of the method and its variants, including the single-image S-COPHY adaptation and ensemble strategies. Section 7 examines limitations and unresolved issues, including hyperparameter sensitivity, class imbalance, conformer representation, reproducibility concerns, and the interpretability gap. Section 8 positions DeepSnap relative to descriptor-based, graph-based, 3D-aware GNN, and other image-based or semi-image-based approaches. Section 9 discusses future directions, including explainable AI methods, robust image-generation protocols, conformer-ensemble extensions, and potential integration with graph-based approaches. Throughout this article, we distinguish between empirical evidence from primary studies and interpretive claims from review articles and flag areas where the evidence base remains incomplete.

This study is intended as a focused narrative review of DeepSnap and its direct derivatives, rather than as a systematic review of all image-based molecular representation methods. To date, DeepSnap has mainly been developed within the author’s research group and by close collaborators. Therefore, a substantial fraction of the primary studies discussed here originates from this research line. To avoid a self-promotional style, this review explicitly places DeepSnap in the broader context of descriptor-based QSAR, graph-based molecular learning, 3D-aware GNNs, and analogous or semianalogous image-based molecular representation methods, including Toxic Colors, Chemception, ImageMol, DEEPScreen, and MolMap/MolMapNet [12,13,14,16,17].

This review also differs from three related 2023 publications by the author’s group: (1) an Elsevier book chapter introduced molecular-image-based deep learning and summarized the DeepSnap concept at its early stage [2]; (2) a review in *Processes* provided a broader overview of deep-learning-based QSAR and parameter optimization [24]; and (3) a review in *Molecules* focused primarily on ensemble learning, molecular descriptor-based machine learning, and DeepSnap–descriptor integration [25]. By contrast, this review provides an updated chronological discussion of the DeepSnap research program from its 2018 origin to its current state, including subsequent developments such as S-COPHY, AI-based Substance Hazard Integrated Prediction System (AI-SHIPS)-related visualization, ADME and toxicological applications, explicit limitations of the method, comparisons with related image-based and graph-based approaches, and the lack of matched-split benchmarking.

## 2. Origin and Rationale of DeepSnap

Quantitative structure–activity relationship (QSAR) modeling has traditionally relied on molecular descriptors—numerical values computed from chemical structures that encode physicochemical, topological, and electronic properties. Although such descriptors have proven effective in many settings, their design requires expert knowledge and their expressiveness is bounded by the assumptions embedded in each descriptor definition. In parallel, circular fingerprints such as ECFP provide a substructure-based encoding that captures local connectivity patterns but largely discards 3D spatial information. A fundamental question therefore arises: can deep learning extract predictive features directly from molecular representations without the intermediary of hand-engineered descriptors?

**Figure 1:**
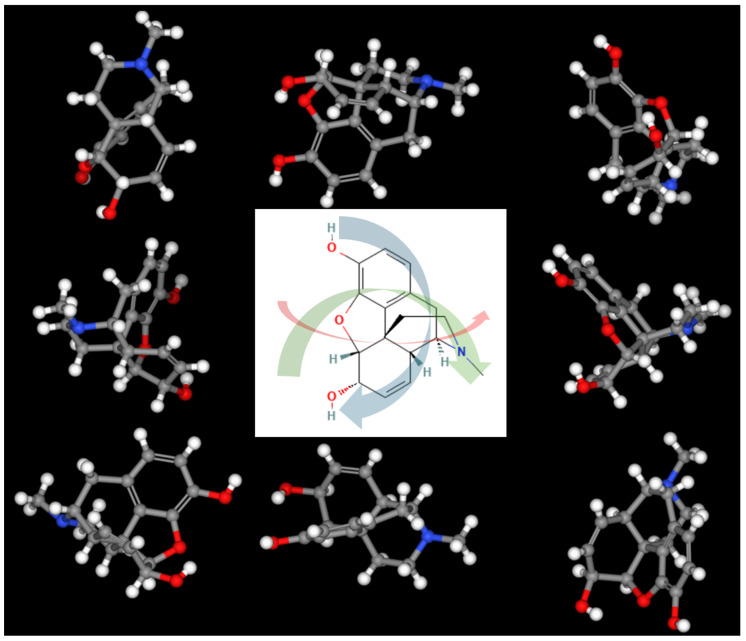
Conceptual illustration of multiangle snapshot acquisition in DeepSnap. A single three-dimensional molecular conformer is viewed from multiple directions, producing distinct two-dimensional projected molecular images. The red, blue, and green arrows schematically indicate rotations around the three coordinate axes. These orientation-dependent snapshots collectively provide the convolutional neural network with structurally diverse visual inputs derived from the same molecule.

The DeepSnap method was introduced in 2018 to address this question [18]. Rather than computing numerical descriptors or fingerprints, DeepSnap converts each molecule into a set of color-coded 3D molecular images that are fed directly into a CNN. Although ball-and-stick renderings were used in the initial and subsequent implementations, the DeepSnap concept is not restricted to a single visualization style. The term “Deep Snap” refers to the omnidirectional snapshot capture procedure: starting from a single 3D conformer, the molecular structure is rotated systematically around the x-, y-, and z-axes at a fixed angular increment and an image is rendered at each orientation [18,26]. The resulting image set provides the CNN with multiple viewpoints of the same molecule, and aggregating per-image predictions across orientations approximates orientation invariance without explicit descriptor computation. Figure 1 illustrates how a single 3D conformer yields distinct 2D snapshots depending on viewing direction.

### 2.1. Inaugural Study

The first DeepSnap study targeted mitochondrial membrane potential (MMP) disruption, one of the endpoints in the Tox21 Data Challenge 2014 [18]. 3D conformations were generated from SMILES strings using CORINA Classic (Molecular Networks GmbH, Nuremberg, Germany; version not specified in the original publication; https://www.mn-am.com/products/corina, accessed on 20 April 2026), and ball-and-stick model images were rendered with Jmol (version not specified in the original publication; http://www.jmol.org/, accessed on 20 April 2026), where each atom type was displayed in a distinct color [18]. Images were saved as 256 × 256 pixel PNG files [18]. With a 45-degree angular increment applied to all three rotation axes, the procedure yielded 512 images per molecule (8 orientations per axis, 8^3^ = 512 combinations) [18]. Moreover, a coarser setting of 90 degrees was tested, producing 64 images per molecule [18].

The deep learning architecture employed was AlexNet [27], implemented within the Caffe framework [18]. Transfer learning with pretrained Caffe models was used in the DeepSnap-DL pipeline from the beginning of the study series, and later methodological papers described this implementation explicitly [28]. The training set comprised 7320 molecules from the Tox21 Data Challenge, divided equally into training and validation subsets, whereas the external test set contained 647 compounds corresponding to the final evaluation data of the challenge [18]. For each molecule, the median of the 512 per-image predicted probabilities was taken as the representative prediction, a design choice that would persist throughout later DeepSnap studies [18].

### 2.2. Performance and Comparators

On the external test set, the DeepSnap model with the 45-degree increment achieved an area under the receiver operating characteristic curve (AUC) of 0.921, an accuracy of 0.836, and a sensitivity of 0.867 [18]. The 90-degree increment model yielded a lower but still competitive AUC of 0.898 [18]. These results were compared against three alternative approaches evaluated on the same dataset: a deep learning model trained on ECFP fingerprints using the H2O framework (AUC 0.888), a random forest (RF) model on ECFP fingerprints (AUC 0.901), and an RF model on 3D molecular descriptors computed by MOE (AUC 0.907) [18]. The DeepSnap model surpassed all three comparators in AUC despite operating without any explicit molecular descriptors or fingerprints.

This result was particularly notable because the Tox21 Data Challenge 2014 winning model for the same MMP disruption endpoint had achieved an AUC of 0.95 [18]. Although DeepSnap did not match this competition benchmark, the difference was 0.029 AUC units and the AlexNet architecture had been used without hyperparameter tuning—the author noted that further optimization could close the remaining gap [18]. Importantly, the winning model relied on conventional molecular descriptors or fingerprints, whereas DeepSnap derived its input solely from rendered molecular images.

However, the comparison was not uniformly favorable across all metrics. The RF model with ECFP achieved a higher accuracy (0.915) and specificity (0.986) than DeepSnap (accuracy 0.836, specificity 0.832), whereas DeepSnap showed substantially higher sensitivity (0.867 vs. 0.350) [18]. This pattern—high sensitivity at the cost of specificity—suggested that the image-based model may encode information complementary to that captured by fingerprints [18].

### 2.3. Conceptual Rationale

The underlying rationale of DeepSnap rests on two premises. First, molecular images generated from prepared 3D conformers encode atom types, bond arrangements, steric shape, and relative atomic positions in a format directly compatible with the feature-extraction capabilities of CNNs originally developed for natural image recognition [2]. The color coding of atom types (e.g., oxygen in red, nitrogen in blue, and carbon in gray) provides the network with chemical identity information within the pixel space [18]. Second, the omnidirectional snapshot procedure mitigates the viewpoint dependence inherent in projecting a 3D object onto a 2D image plane; by aggregating predictions from multiple orientations, the model approximates a viewpoint-invariant representation [18].

This image-based strategy differs from GNNs, which represent molecules as mathematical graphs with atoms as nodes and bonds as edges [2,24]. However, the contrast should not be framed as a simple distinction between 2D GNNs and 3D DeepSnap input because 3D-aware GNNs such as SchNet explicitly incorporate molecular geometry [11]. The more precise distinction is representational: DeepSnap learns from pixel-level visual features rendered from prepared 3D conformers, whereas graph-based methods learn from topological or geometric message passing. Whether DeepSnap provides advantages over topology-based GNNs, 3D-aware GNNs, or other image-based approaches under matched datasets, splits, and evaluation protocols remains unresolved [24].

### 2.4. Early Recognition of Limitations

The inaugural publication also identified several limitations that would shape subsequent research directions. The computational cost of generating hundreds of images per molecule was acknowledged as “decidedly more costly” than descriptor-based methods [18]. The reliance on a single conformer from CORINA Classic, without molecular dynamics or force-field refinement, was noted as a potential source of error for flexible molecules [18]. In addition, only a single toxicological endpoint had been tested, leaving the generalizability of the approach undemonstrated [18]. These limitations set the agenda for the systematic parameter optimization, architecture upgrades, and multiendpoint applications that followed in subsequent years.

## 3. Core Rendering and Learning Pipeline

The prediction performance of DeepSnap depends on the interplay of rendering parameters, CNN architecture, hyperparameter tuning, and postprocessing strategies. Over the course of several studies, these components were systematically optimized, first within a DIGITS/Caffe-based pipeline and later within a redesigned TensorFlow/Keras system.

### 3.1. Rendering Parameter Optimization

The DeepSnap image-generation procedure is governed by six rendering parameters: molecules per SDF (MPS), zoom factor (ZF), atom size (AT), bond radius (BR), minimum bond distance (MBD), and bond tolerance (BT). Matsuzaka and Uesawa conducted a systematic grid search over each of these parameters for the CAR agonist endpoint, revealing that each parameter exhibited a quadratic relationship with validation loss (R-squared greater than 0.90 for all six) [28]. The optimal values identified were MPS: 150, ZF: 80%, AT: 22%, BR: 20 milliangstroms, MBD: 0.4 angstroms, and BT: 0.8 angstroms [28]. These results demonstrated that rendering parameters are not arbitrary but have well-defined optima that can be located through systematic tuning. The study also showed that multicolor atom representations yielded lower validation loss than monotone gray or monotone white renderings (validation loss 0.442 versus 0.468 and 0.467, respectively), though a two-color scheme (gray plus white) achieved an intermediate validation loss of 0.437 [28].

The optimization of DeepSnap image-generation parameters should be interpreted as the optimization of the molecular representation supplied to the CNN, rather than the optimization of physical molecular variables. Parameters such as the pixel size, atom-color scheme, background color, molecular orientation, and viewing angle influence how molecular structures are projected onto image space. Adjusting these parameters may improve the machine readability of structural patterns, but they also expand the effective model-selection space and require validation steps to separate parameter selection from final testing.

### 3.2. First-Generation Pipeline

**Figure 2:**
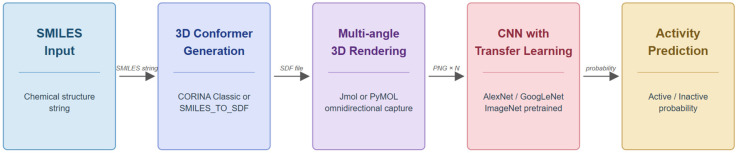
Schematic of the DeepSnap pipeline. SMILES strings are converted to three-dimensional conformers, rendered as color-coded ball-and-stick images from multiple viewing angles, and classified by a CNN with transfer learning from ImageNet.

The original DeepSnap pipeline consisted of four discrete steps. Molecular structures were first cleaned using MOE 2018 (MOLSIS Inc., Tokyo, Japan; Chemical Computing Group, Montreal, QC, Canada), then converted to 3D conformations by CORINA Classic (Molecular Networks GmbH, Nuremberg, Germany; version not specified in the cited original studies; https://www.mn-am.com/products/corina, accessed on 20 April 2026), and finally rendered as 256 × 256 pixel ball-and-stick PNG images using Jmol (version not specified in the cited original studies; http://www.jmol.org/, accessed on 20 April 2026) [28,29]. Deep learning was performed using AlexNet within the NVIDIA DIGITS (version 4.0.0; NVIDIA, Santa Clara, CA, USA) interface running on the Caffe framework (version not specified in the cited original studies), with transfer learning from ImageNet-pretrained weights [28]. Training typically ran for 30 epochs with SGD at a base learning rate of 0.01, and the epoch with the lowest validation loss was selected for test prediction [28]. Figure 2 summarizes the first-generation DeepSnap workflow, from SMILES input and 3D conformer generation to multiangle molecular image rendering and CNN-based prediction.

### 3.3. Transition from AlexNet to GoogLeNet

A direct comparison of AlexNet and GoogLeNet on the CAR agonist endpoint showed that GoogLeNet achieved an AUC of 0.886 versus 0.857 for AlexNet under the same pre-CORINA-optimization conditions [29]. The adoption of GoogLeNet [30], a 22-layer Inception-based CNN pretrained on the ILSVRC 2012 dataset (1.2 million images, 1000 classes), became standard from this point forward [29,31]. All subsequent DeepSnap studies employed GoogLeNet with transfer learning from ImageNet-pretrained weights, run through DIGITS/Caffe in the first-generation system [31,32].

### 3.4. CORINA Wash Conditions

The quality of 3D input structures proved to be a dominant factor in prediction performance. Ten wash combinations (three protonation states crossed with three coordinate systems plus one mixed condition) were evaluated for the CAR endpoint; the neut_CORINA condition (neutralization followed by CORINA Classic conformation generation) achieved the highest mean AUC of 0.995 across nine learning rates [29]. However, when the same wash comparison was performed for the PR antagonist endpoint, the domi_3D condition yielded a best AUC of 0.9971, whereas none_2D performed worst [31]. This finding that the optimal conformational preparation varies across endpoints was explicitly noted by the authors and represents an important caveat against adopting a single universal wash protocol [31].

### 3.5. Angle Increment as Key Hyperparameter

The viewing-angle increment, which determines the number of snapshot images generated per molecule and the diversity of perspectives captured, emerged as one of the most influential hyperparameters. For the CAR endpoint, 92 different angle configurations were scanned, with the best single-angle configuration (176 degrees) yielding an AUC of 0.910 before wash optimization [29]. The CAR model demonstrated robustness across data split ratios under the neut_CORINA wash condition: at the 176-degree angle, 1:1:1 and 4:4:1 splits achieved comparable AUC values (0.998 ± 0.002 and 0.999 ± 0.001, respectively), with corresponding MCC values of 0.954 ± 0.026 and 0.966 ± 0.023 [29]. For the PR endpoint, angles from 120 to 300 degrees all achieved AUC values of 0.996 or higher, whereas the 360-degree condition (a single image) dropped to an AUC of 0.855 at 1:1:1, demonstrating that multiangle snapshots are essential for high performance [31]. Even with as few as four pictures at 280 degrees, the CAR model retained an AUC of 0.999 and an MCC of 0.967 [29].

### 3.6. Second-Generation Pipeline

In 2021, the DeepSnap system underwent a comprehensive migration. The DL framework was changed from DIGITS/Caffe (DIGITS version 4.0.0; Caffe version not specified in the cited original studies) to TensorFlow with Keras (versions not specified in the cited original studies), the rendering software from Jmol (version not specified; http://www.jmol.org/, accessed on 20 April 2026) to PyMOL (Schrödinger, LLC, New York, NY, USA; version not specified in the cited original studies; https://pymol.org, accessed on 20 April 2026), and the 3D structure generation from the MOE/CORINA Classic pipeline to the SMILES_TO_SDF program (version not specified in the cited original studies) [20]. The Merck Molecular Force Field was used in the PyMOL-based pipeline for conformational optimization [33]. These changes were integrated into a one-step sequential pipeline that improved throughput by automating all stages from SMILES input to statistical evaluation [20]. However, the initial 59-MIE model set constructed with the new system achieved a mean ROC–AUC of 0.818 on validation, compared with 0.884 for a previous 35 nuclear receptor model set built with DIGITS, indicating that the system migration introduced a performance trade-off that required further optimization [20]. Parallel comparisons on individual endpoints showed that DIGITS outperformed the TensorFlow/Keras system in mean AUC across all three endpoints tested, though systematic per-endpoint optimization of learning rate and batch size substantially improved the TensorFlow/Keras results [33].

### 3.7. Background Color Optimization

Background color was explored as an additional rendering variable. In the first-generation pipeline, six colors (white, red, yellow, green, blue, and black) were tested for the PR endpoint; white and black produced considerably lower performance at the 360-degree single-image condition, while all other colors performed comparably at 300 degrees [31]. The second-generation system expanded this search to more than 20 colors. For the PPARγ agonist endpoint, wheat and Grey90 emerged as the best standard and pastel colors on the test set (ROC–AUC of 0.931 and 0.935, respectively), while for the aromatase antagonist endpoint, tv_orange yielded a test ROC–AUC of 0.905 [20]. The authors noted that background color effects may relate to the colors present in the ImageNet pretraining data [20].

### 3.8. Learning Rate, Batch Size, and Epoch Selection

Learning rate and batch size required per-endpoint optimization. For the aryl hydrocarbon receptor (AhR) activation endpoint, the NAG solver with a learning rate of 0.0025 and batch size of 37 achieved the lowest validation loss of 0.1466 [32]. For PR antagonist prediction, the learning rate range 0.01 to 0.001 was optimal, and performance generally decreased with increasing batch size; among six solver types tested, RMSprop showed the best performance and AdaDelta the worst performance among the six solvers tested [31]. In the second-generation system, even wider hyperparameter sweeps were conducted: for the GR antagonist endpoint, 39 learning rates and 84 batch sizes were evaluated, ultimately achieving a test ROC–AUC of 0.983 at batch size 125 [33]. Epoch selection across all studies relied on the lowest validation loss criterion, with 30 epochs as the standard training duration and early stopping introduced in the TensorFlow/Keras system [20,33].

### 3.9. Aggregation, Cut-Off Determination, and Model Validation

Because DeepSnap generates multiple images per molecule, predictions must be aggregated across viewing angles. Across all studies, the median of per-image predicted probabilities was adopted as the representative prediction value for each molecule [28,29,31,32]. For binary classification, the optimal probability cut-off was determined using the Youden index [20,32,33].

Model validity was assessed through permutation tests in which class labels were randomly shuffled before training. For the CAR endpoint, the permutation test yielded a mean AUC of 0.553 (standard deviation 0.007, ten repetitions), far below the true-label AUC of 0.764, confirming that the model captured genuine structure–activity relationships rather than noise [28]. Similar random-label controls for the PR antagonist endpoint produced permutation AUC values of 0.519 to 0.527 [31], and for the AhR endpoint a permutation AUC of 0.539 [32]. The consistently near-chance permutation AUC values across multiple endpoints provide important evidence that DeepSnap-DL models are learning meaningful chemical features from the molecular images.

## 4. Applications to Toxicological Targets

The primary testing ground for DeepSnap-DL has been the Tox21 10K compound library, a publicly available collection of 7000–10,000 chemicals screened across multiple in vitro bioassays under the interagency Tox21 program. Throughout the DeepSnap series, binary activity was defined using a PubChem activity score threshold of 40 or above as active, with scores below 40 classified as inactive [19,29]. This standardized criterion replaced the more permissive threshold of score greater than zero used in the earliest parameter optimization study [28]. The Tox21 datasets are characterized by pronounced class imbalance, with active compound fractions typically ranging from below 1% to approximately 20% across different endpoints [19,20].

The first application of DeepSnap targeted MMP disruption, one of the Tox21 Data Challenge 2014 endpoints. Using AlexNet with 512 images per molecule at 45-degree rotation increments, the model achieved an AUC of 0.921 on the external validation set of 647 compounds [18]. This result compared favorably with descriptor-based alternatives tested on the same data: a deep learning model using ECFP fingerprints yielded an AUC of 0.888, and RF models with ECFP or 3D MOE descriptors achieved AUCs of 0.901 and 0.907, respectively [18]. However, the Tox21 Data Challenge 2014 winning model for this endpoint had achieved an AUC of 0.95, indicating a gap between the initial DeepSnap implementation and state-of-the-art competition entries [18].

Subsequent studies applied DeepSnap-DL to individual nuclear receptor targets from the Tox21 library, progressively refining the methodology through GoogLeNet adoption, conformer-preparation optimization, viewing-angle selection, and corrected activity-threshold definitions [29,31]. The CAR agonist and PR antagonist studies reported very high ROC–AUC values under their respective study-specific datasets, image-generation settings, and validation protocols, as summarized in Table 1. These results supported the feasibility of DeepSnap for molecular-image-based QSAR; however, they should not be interpreted as near-perfect or deterministic prediction without further external and matched-split validation.

A notable departure from the Tox21 data occurred with the prediction of AhR activation using a small in-house dataset of 201 compounds measured by a reporter gene assay [32]. Despite the limited dataset size, DeepSnap-DL achieved an AUC of 0.959 at the optimal activity threshold, substantially exceeding the best conventional machine learning comparator (XGBoost, AUC: 0.724) tested on the same data [32]. This result demonstrated that the molecular image approach could be effective beyond large public screening libraries, although the small sample size warrants cautious interpretation.

The scope of DeepSnap-DL was then expanded to 35 nuclear receptor agonist and antagonist endpoints from the Tox21 library in a single comprehensive study [19]. Using GoogLeNet with the DIGITS framework, a 176-degree angle yielding 27 images per compound, and a uniform set of hyperparameters across endpoints, the study showed that DeepSnap could be scaled beyond single-endpoint optimization. Detailed endpoint-level performance metrics, including the mean AUC and threshold-dependent metrics, are summarized in Table 1. The strong class imbalance across these endpoints was reflected in modest F-measure and MCC values, underscoring that high ranking performance does not eliminate the practical difficulty of threshold-dependent minority-class prediction [19].

The transition to an improved system based on TensorFlow and Keras, with PyMOL replacing Jmol for rendering and a new 3D structure generation pipeline (SMILES_TO_SDF), enabled construction of 59 MIE models encompassing nuclear receptor and stress response pathway targets [20]. The initial 59-model run produced lower mean performance than the earlier DIGITS-based 35-NR model set, a difference attributed to system migration and the need for further per-endpoint optimization [20]. Separately, MORDRED descriptor-based XGBoost models covering 58 of the 59 MIE endpoints (VDR_ago excluded owing to insufficient active compounds under the criterion of a score ≥40) achieved comparable average performance [34].

Per-endpoint hyperparameter optimization of the TensorFlow/Keras system was subsequently demonstrated for three MIE targets [33]. Systematic tuning of learning rate, batch size, angle, and data split ratio yielded AUCs of 0.983 for GR antagonist, 0.934 for TRHR agonist, and 0.925 for TGFβ antagonist on the test set [33]. These results substantially exceeded the mean performance of the initial 59-model run and demonstrated that the TensorFlow/Keras system could match or approach DIGITS-level performance when optimized on a per-endpoint basis (Table 1).

Beyond the Tox21 in vitro assay framework, DeepSnap-DL was applied to acute oral toxicity prediction using the Collaborative Acute Toxicity Modeling Suite (CATMoS) dataset [22,35]. This study combined image-based DeepSnap predictions with descriptor-based machine learning and reported ensemble and consensus models for LD50 classification. The CATMoS collaborative consensus model served as an important contextual comparator; however, differences in model-development workflow, coverage, and validation design mean that the results should be interpreted as contextual rather than as a matched head-to-head comparison. Detailed values are provided in Table 1.

In addition, the DeepSnap approach was extended to S-COPHY, a Shiseido-developed system name for a single-image DeepSnap-derived model that classifies compounds as cosmetics- or pharmaceutical-related based on 3D molecular images; the original publication does not provide a formal acronym expansion [23]. This application classified compounds based on structural similarity, using a single molecular image per compound selected from 27 candidates by maximum pixel occupation, with AlexNet implemented in MATLAB R2021a (MathWorks, Natick, MA, USA) [23]. S-COPHY provided a peer-reviewed example of Grad-CAM visualization for a DeepSnap-type model, showing that the CNN focused on pharmaceutical-like structural motifs within the molecular image [23].

A recurring challenge across all DeepSnap-DL toxicological applications has been class imbalance. In the 59-MIE study, active compound fractions ranged from 0.08% (TGFβ agonist) to 21.6% (PXR agonist), with a mean of 4.79% [20]. Threshold-dependent metrics such as MCC and F-measure reflected the difficulty of classifying the minority active class. For example, the 35-NR study reported a mean F-measure of 0.309 and a mean MCC of 0.354 across endpoints with a mean active fraction of only 3.7% [19]. Similarly, the TRHR agonist endpoint (0.87% active compounds) yielded an MCC of 0.200 after optimization [33]. These observations underscore that threshold-dependent classification of minority-class compounds remains challenging in the Tox21 context, a limitation shared with other modeling approaches applied to these datasets.

**Table 1:** Chronological summary of representative DeepSnap and DeepSnap-related studies. Endpoint-level performance metrics are shown here to avoid repeated numerical reporting in the main text. Because datasets, splits, preprocessing procedures, image-generation settings, and evaluation protocols differed across studies, these values should not be interpreted as direct head-to-head comparisons unless explicitly stated. Abbreviations: CB, CatBoost; CL, clearance; DIGITS, NVIDIA Deep Learning GPU Training System; DL, deep learning; ens., ensemble; cons., consensus; LGBM, LightGBM; MD, molecular descriptors; RF, Random Forest; TF, TensorFlow; XGB, XGBoost.

Year	Ref.	Endpoint	Dataset (N)	Pipeline	Best Result	Comparator	Key Limitation
2018	[18]	MMP disruption	Tox21 (7967)	AlexNet/DIGITS	AUC 0.921	RF + 3D desc 0.907	Single endpoint; no HP tuning
2019	[28]	CAR param. optim.	Tox21 (9523)	AlexNet/DIGITS	AUC 0.791	RF + MOE 0.749	Non-standard threshold; single split
2019	[29]	CAR agonist	Tox21 (7141)	GoogLeNet/DIGITS	AUC 0.999	XGB 0.889; RF 0.884	Near-ceiling AUC; class imbalance
2020	[31]	PR antagonist	Tox21 (7582)	GoogLeNet/DIGITS	AUC 0.999	CB 0.894; LGBM 0.893	Wash varies by target; imbalance
2020	[32]	AhR (in-house)	In-house (201)	GoogLeNet/DIGITS	AUC 0.959	XGB 0.724; RF 0.716	Small N; no wash optimization
2020	[19]	35 NR models	Tox21 (mean 7262)	GoogLeNet/DIGITS	Mean AUC 0.884	Tox21 Challenge (3/4 exceeded)	Two replicates; class imbalance
2021	[20]	59 MIE models	Tox21 (mean 9699)	GoogLeNet/TF-Keras	Mean AUC 0.818	Prior DIGITS system	Underperforms DIGITS; NF-κB failed
2021	[21]	Rat CL classif.	In-house (1545)	GoogLeNet/DIGITS + ens.	Ens. AUC 0.943	RF + MD 0.883	Consensus coverage 69%
2022	[33]	GR/TRHR/TGFβ	Tox21 (7537–7662)	GoogLeNet/TF-Keras	GR AUC 0.983	DIGITS GR 0.910	TRHR MCC 0.200; 3 endpoints only
2022	[36]	Rat CL regression	In-house (1545)	DL prob. + AutoML	R^2^ 0.736	MD-only R^2^ 0.649	DL alone R^2^ 0.359; private data
2023	[22]	LD50/BBBP/CL path.	CATMoS (11,886) etc.	GoogLeNet/DIGITS + ens.	Cons. BAC 0.916	CATMoS 32 orgs 0.87	Coverage 77–86%; DL < CATMoS
2024	[23]	Cosmetics vs. pharma	PubChem etc. (2754)	AlexNet/MATLAB	AUC 0.935	None	Ext. pred. rate 46%; regulatory

## 5. Applications to ADME Parameters

Although the initial applications of DeepSnap-DL focused on toxicological targets from the Tox21 library and related assays, a parallel line of investigation extended the approach to ADME and pharmacokinetic (PK) parameters. This expansion was motivated by the practical importance of early PK prediction in drug discovery and by the hypothesis that molecular image-based features might capture structural information relevant to processes governed by drug–transporter interactions and metabolic clearance, not solely receptor binding.

The first ADME application of DeepSnap-DL targeted the binary classification of rat clearance (CL), a parameter that had historically proven difficult to predict with conventional machine learning methods [21]. Using an in-house dataset of 1545 compounds from Japan Tobacco Inc., the study constructed two independent modeling pipelines: a DeepSnap-DL model based on GoogLeNet and 3D molecular images, and a descriptor-based RF model selected by DataRobot from conventional molecular descriptors [21]. The principal endpoint-level metrics are summarized in Table 1.

The more consequential finding was that combining the two approaches produced substantial improvements beyond either method alone. An ensemble model averaged the predicted probabilities from DeepSnap-DL and the descriptor-based model, whereas a consensus model retained predictions only when both methods agreed on the class assignment [21]. This consensus approach improved threshold-dependent performance but reduced the number of evaluable compounds, representing a trade-off between prediction confidence and coverage. Mamada et al. also discussed independently published rat clearance QSAR models as external context while noting that direct comparison was difficult because the compound sets and modeling protocols differed [21].

Having established the value of the combination approach for CL classification, a subsequent study addressed the regression prediction of continuous CL values using the same 1545-compound dataset [36]. Rather than averaging probabilities as in the ensemble classification model, this study introduced a conceptually distinct strategy: the DeepSnap-DL classification probability was appended as an additional descriptor to the conventional molecular descriptor set, and the augmented feature matrix was used to train regression models via DataRobot [36]. This probability-as-descriptor approach was validated using a five-pattern cross-dataset design, in which the compounds were divided into five equal subsets and each pattern used four subsets for training and one for testing [36]. The combination model achieved a mean test R-squared of 0.736 with a mean RMSE of 0.265, compared with R-squared of 0.649 and RMSE of 0.306 for the descriptor-only regression [36]. The improvement was consistent across all five data partitions, with test R-squared values ranging from 0.710 to 0.769 for the combination model versus 0.625 to 0.669 for the descriptor-only model [36].

Feature importance analysis of the combination regression model revealed the dominance of the image-derived probability feature. The DeepSnap-DL prediction probability had an average effect of 1.000 (normalized to the top feature), whereas the next most important descriptor (BCUT_SLOGP_0, a lipophilicity-related descriptor) had an average effect of only 0.107 [36]. This pronounced gap indicated that the image-based classification probability encoded structural information largely orthogonal to conventional physicochemical descriptors. Notably, the DeepSnap-DL probability alone explained only R-squared of 0.359 of the variance in log(CL), suggesting that its value lies primarily in its complementarity with descriptor-based features rather than in its standalone predictive power [36].

The generalizability of the ensemble and consensus combination strategies was subsequently tested on two additional ADME-related endpoints alongside the LD50 toxicity target [22]. For blood–brain barrier penetration (BBBP) and clearance-pathway classification, the ensemble models again improved on the individual image-based and descriptor-based components, whereas consensus models improved balanced accuracy at the cost of reduced coverage. The published study compared these results with independently reported BBBP, CATMoS, and clearance-pathway models; however, these were indirect comparisons because of mismatches in the datasets, compound selections, splitting strategies (including time-split validation as an estimate of prospective performance), endpoint definitions, and metrics [22,35,37,38,39,40,41].

The robustness of estimated aqueous solubility (ESOL) prediction was also examined using the probability-as-descriptor approach on 1128 compounds from the MoleculeNet benchmark [36]. Here, the improvement was modest: the combination model achieved a mean R-squared of 0.950 versus 0.943 for the descriptor-only model [36]. This smaller gain, compared with the larger improvement observed for rat CL, suggests that the combination approach provides the greatest benefit when conventional descriptors alone are insufficient to capture the relevant structure–property relationships. When descriptor-based models already perform well, as with ESOL, the marginal contribution of image-derived features diminishes.

Across the tested ADME and PK endpoints, image-based DeepSnap predictions and descriptor-based predictions consistently provide complementary information [21,22,36]. However, compared with some Tox21-based nuclear receptor applications, descriptor-based models yielded higher standalone AUC values than DeepSnap-DL for several ADME-related tasks [22]. This suggests that the primary value of DeepSnap-DL in the ADME domain lies not in standalone superiority but in contributing complementary structural information that strengthens ensemble, consensus, or probability-as-descriptor models.

Several limitations should be noted. The rat CL studies relied on a proprietary dataset from a single pharmaceutical company, limiting independent reproduction [21,36]. The CL pathway dataset contained only 636 compounds, with a relatively small test set [22]. The consensus model’s requirement for agreement between methods introduces a coverage–accuracy trade-off that may constrain its applicability in early drug screening, where comprehensive evaluation of all candidate compounds is often preferred [21,22]. Furthermore, the DeepSnap-DL angle was fixed at 145 degrees for ADME applications based on the initial CL study, without full per-endpoint rendering optimization, which may have reduced standalone image-model performance [22]. Despite these caveats, the ADME studies collectively support the complementarity between molecular image features and conventional descriptors.

## 6. Technical Evolution and Variants

**Figure 3:**
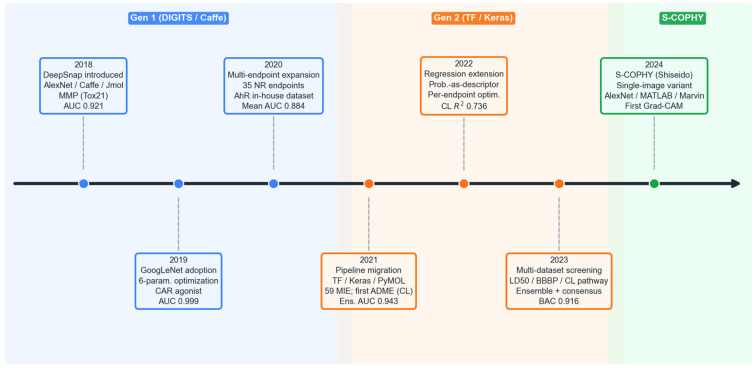
Timeline of DeepSnap development (2018–2024). Blue: first-generation DIGITS/Caffe/Jmol pipeline; orange: second-generation TensorFlow/Keras/PyMOL pipeline; green: S-COPHY industrial variant. Milestone annotations indicate key publications and methodological advances.

Since its introduction in 2018, the DeepSnap-DL system has undergone substantial technical changes affecting the deep learning framework, the 3D structure generation pipeline, the molecular rendering software, and the strategies used for combining predictions across models. These modifications can be organized into two broad pipeline generations, a single-image variant (S-COPHY), combination strategies that integrate image-based and descriptor-based predictions, and emerging work on model interpretability (Figure 3).

The first generation of DeepSnap-DL (2018–2020) was built on the NVIDIA DIGITS with Caffe as the underlying framework, running on four Tesla V100 GPUs [29,31]. 3D molecular structures were generated through a pipeline in which SMILES strings were processed by MOE for protonation and desalting, and then optimized by CORINA Classic to produce SDF files [29]. The open-source Java-based viewer Jmol rendered these structures as ball-and-stick images at 256 × 256 pixel resolution [28]. The CNN architecture transitioned from AlexNet in the earliest study [18] to GoogLeNet beginning with the 2019 CAR agonist work [29]. Transfer learning from ImageNet-pretrained weights was employed in the first-generation DIGITS-based studies [24,28]. This first-generation system produced the highest reported performances in the DeepSnap series; however, these values should be interpreted in the context of endpoint-specific parameter optimization, class imbalance, and limited matched external benchmarking.

The second generation, introduced in 2021, migrated the deep learning framework from DIGITS/Caffe (DIGITS version 4.0.0; Caffe version not specified in the cited original studies) to TensorFlow and Keras (versions not specified in the cited original studies), replaced Jmol with the Python-based renderer PyMOL (Schrödinger, LLC, New York, NY, USA; version not specified in the cited original studies; https://pymol.org, accessed on 20 April 2026), and substituted the MOE/CORINA workflow with the SMILES_TO_SDF program (version not specified in the cited original studies) for 3D structure generation [20]. Critically, these components were integrated into a one-step sequential pipeline that automatically executed 3D generation, snapshot rendering, model training, and statistical evaluation [20]. The GoogLeNet architecture was retained, and early stopping based on validation loss was introduced as a regularization strategy [20]. This redesign prioritized throughput and reproducibility over peak accuracy. When applied to 59 MIE models from the Tox21 library, the second-generation system achieved a mean validation AUC of 0.818, compared with the mean AUC of 0.884 reported for 35 nuclear receptor models in the first-generation system [19,20]. Direct parallel comparisons on individual endpoints showed that DIGITS consistently outperformed TensorFlow/Keras in mean AUC across all angles and splits tested [33]. For example, on the glucocorticoid receptor antagonist endpoint, the DIGITS system achieved a mean validation AUC of 0.856 versus 0.832 for the Python system [33]. However, with systematic per-endpoint optimization of learning rates and batch sizes, the TensorFlow/Keras system could reach high individual-endpoint performance, as demonstrated by a test AUC of 0.983 for GR antagonist after batch size optimization [33].

An intriguing phenomenon observed in the second-generation system was a two-peak pattern in performance as a function of rotation angle: TensorFlow/Keras models showed performance peaks at approximately 180 degrees and 360 degrees, a pattern not observed in DIGITS models [20]. This observation was reproduced across multiple endpoints [33] and may reflect differences in how the two frameworks handle image preprocessing or weight initialization, though the precise cause remains unidentified. An important caveat in interpreting the performance gap between generations is that the system migration changed multiple components simultaneously—the DL framework, the 3D structure generator, and the renderer—making it impossible to isolate the contribution of any single factor to the observed decrease in mean performance [20].

The S-COPHY variant, developed with Shiseido, departed from the multiangle snapshot paradigm [23]. S-COPHY is a system name rather than a formally expanded acronym in the original publication. Instead of generating multiple images per compound and aggregating predictions across angles, S-COPHY generated 27 candidate images by rotating the 3D structure at 120-degree increments on each axis and selected a single image per compound based on the maximum percentage of pixels occupied by the molecular structure [23]. The implementation used AlexNet rather than GoogLeNet, MATLAB R2021a (MathWorks, Natick, MA, USA) as the deep learning framework, and Marvin (ChemAxon, Budapest, Hungary; version not specified in the original publication; https://chemaxon.com/marvin, accessed on 20 April 2026) for 3D structure generation [23]. Although the task differs from Tox21-based activity prediction, this result demonstrated that a single well-chosen molecular image can carry useful structural information for classification, substantially reducing the computational cost of the multiangle approach.

Beyond modifications to the image generation and classification pipeline, the DeepSnap series introduced strategies for combining image-based predictions with conventional descriptor-based machine learning. For classification tasks, ensemble and consensus models combining DeepSnap-DL and descriptor-based predictions improved performance or confidence relative to either representation alone (Table 1) [21,22]. For regression tasks, a conceptually distinct probability-as-descriptor strategy was introduced, in which the DeepSnap-DL classification probability was appended as a feature to the conventional descriptor set and used to train regression models [36]. This image-derived probability emerged as the most important feature in the clearance regression model, supporting the interpretation that DeepSnap-derived image features can encode information complementary to conventional descriptors [36].

Efforts toward interpreting DeepSnap-DL predictions have advanced through multiple channels, although the area remains at an early stage. The S-COPHY study provided Grad-CAM [42] visualizations for a molecular image-based CNN, demonstrating that the model’s attention concentrated on specific pharmacophoric substructures in compounds predicted as pharmaceuticals [23]. Within the main DeepSnap series, Grad-CAM analysis was reported to confirm that the CNN detected chemical structure features within the molecular images, although the results were not shown [20]. In the Ministry of Economy, Trade and Industry (METI) AI-SHIPS project, DeepSnap was implemented in the integrated toxicity prediction system with CAM-family visualization functionality, including ScoreCAM and Guided Grad-CAM options [43,44]. A book chapter discussed the applicability of additional explainable AI techniques—including LIME, SHAP, Anchor, and integrated gradients—to molecular image-based models [2]. In the DeepSnap publications and AI-SHIPS project materials covered in this review, the documented explainability implementations are CAM-family methods, whereas practical applications of LIME, SHAP, Anchor, and integrated gradients to DeepSnap molecular images have not yet been described.

## 7. Limitations and Unresolved Issues

Despite the strong prediction performance reported across multiple endpoints, the DeepSnap-DL approach exhibits several methodological limitations and unresolved issues that warrant critical examination. The most important issues include endpoint-specific parameter sensitivity, the interpretation of near-ceiling ROC–AUC values, class imbalance and validation design, static conformer representation, reproducibility, external benchmarking, and interpretability.

### 7.1. Parameter Sensitivity and Nonphysical Image-Rendering Hyperparameters

A persistent conclusion throughout the DeepSnap literature is that optimal rendering and training parameters are endpoint-dependent and cannot be transferred across targets. Performance can be affected by the six rendering parameters described above, as well as by viewing-angle increments, background color, learning rate, batch size, and epoch selection [20,28,29,31,33]. These parameters are not physical molecular properties but are representation hyperparameters that determine how the molecular geometry and atom-type information are encoded into CNN-readable pixel space. Such dependence is somewhat expected because CNNs learn from image-level spatial patterns. However, strong endpoint-specific sensitivity also raises concerns about over-tuning if image-rendering parameters are repeatedly optimized without strict separation between model selection and final testing.

Therefore, a high performance obtained after image-parameter optimization reflects the performance under the reported representation settings and should not be interpreted as evidence that the underlying chemical relationship has been fully captured. Future studies should report all image-generation settings, evaluate sensitivity across reasonable parameter ranges, and validate selected settings using independent test sets, scaffold splits, or matched benchmark protocols.

### 7.2. Interpretation of Near-Ceiling ROC–AUC Values

Several DeepSnap studies reported very high ROC–AUC values for selected endpoints, including nuclear receptor models, such as those used for CAR and PR antagonist prediction [29,31]. High ROC–AUC values indicate strong ranking or discrimination performance under the specific datasets, molecular-image generation settings, model architectures, and validation protocols used in the original studies. However, they should not be interpreted as evidence of deterministic prediction, universal generalizability, or general superiority over other molecular representation approaches. Near-ceiling ROC–AUC values can reflect endpoint-specific optimization, a favorable training/testing split, class imbalance, structurally similar compounds across splits, or strong assay-specific patterns in the dataset.

This review was based on the metrics reported in the original publications and did not recalculate PR-AUC, calibration, or confusion-matrix-based metrics when prediction scores were not available. Therefore, the ROC–AUC should be treated as one component of model evaluation rather than as an overall measure of predictive reliability. This is particularly important for imbalanced datasets, for which precision–recall analysis can provide additional information about performance on the positive class [45]. Future DeepSnap studies should report complementary metrics, including PR-AUC, balanced accuracy, MCC, sensitivity, specificity, confusion matrices, and calibration measures. Furthermore, robustness should be evaluated under repeated random splits, scaffold splits, and independent external test sets.

### 7.3. Statistical Evaluation Practices, Class Imbalance, and Matched Replication

The Tox21 datasets used in many DeepSnap studies are severely imbalanced. The mean active fraction across 35 nuclear receptor models was low, and the 59-MIE model set similarly contained endpoints with very small positive-class fractions [19,20]. Under such imbalance, threshold-dependent metrics such as F-measure and MCC were modest, even when the ROC–AUC values were high. The validation design also varied across studies, with some studies using few replicates or single training/testing splits. Several DeepSnap-related studies, especially those by Mamada et al., compared their results with independently published QSAR models or benchmark efforts, including prior clearance models, CATMoS, BBBP models, and clearance-pathway models [21,22,35,37,40,41]. These comparisons provide useful external context but were generally indirect comparisons because datasets, compound selections, endpoint definitions, splitting strategies, or evaluation metrics differed. Therefore, independent external replications of the DeepSnap pipeline under matched datasets and splits, and common evaluation protocols remain limited.

### 7.4. Representative Conformers and Lack of Conformational Ensemble Sampling

DeepSnap uses conformer-derived molecular images; however, the multiview images generated in most DeepSnap studies represent different projections of a prepared 3D structure rather than a molecular-dynamics or force-field-sampled conformational ensemble. Multiple viewing angles are useful within the DeepSnap framework; however, they should not be considered a substitute for conformational sampling. A single or representative conformer may be insufficient to describe flexible molecules in solution, especially when the endpoint depends on conformational equilibria, ligand–protein binding adaptation, transport processes, metabolism, or solvent-dependent molecular properties. Future studies should evaluate whether incorporating multiple low-energy conformers, Boltzmann-weighted conformer ensembles, or molecular-dynamics-derived snapshots improves predictive robustness and external generalizability.

### 7.5. Reproducibility, Data Availability, and System Migration

Several DeepSnap studies relied on proprietary, nonpublic datasets that preclude independent reproduction, including the AhR activation dataset and the rat clearance datasets [21,32,36]. Although the Tox21-based studies drew on publicly available data, early 3D structure generation pipelines relied on commercial software such as MOE and CORINA Classic, whose exact behavior may vary across versions. The transition from the original DIGITS/Caffe/Jmol system to TensorFlow/Keras/PyMOL also changed multiple components simultaneously—the deep learning framework, molecular renderer, and 3D structure generator—making it difficult to isolate the source of performance differences observed after system migration [20,33].

### 7.6. Interpretability and Comparison with Alternative Architectures

Throughout most of the DeepSnap series, CNN-based prediction has operated as a black box. Peer-reviewed Grad-CAM visualization using S-COPHY was reported, and CAM-family visualization was implemented using AI-SHIPS [23,42,43,44]. However, a lack of systematic atom-level attribution from pixel-space gradients is observed. In addition, no DeepSnap study has performed a controlled head-to-head comparison with topology-based GNNs, 3D-aware GNNs, or other image-based molecular representation methods under matched datasets, conformer-generation procedures, training/validation/testing splits, and evaluation metrics. Addressing this benchmarking gap is critical for interpreting DeepSnap’s current position in molecular property prediction.

## 8. Position Relative to External Methods

### 8.1. Caution Regarding Cross-Study Comparisons

No matched-split head-to-head benchmarking study has yet compared DeepSnap with descriptor-based QSAR models, topology-based GNNs, 3D-aware GNNs, or other image-based molecular prediction methods using identical datasets, preprocessing procedures, conformer-generation settings, molecular-image rendering parameters, training/validation/testing splits, hyperparameter-search spaces, and evaluation metrics. Therefore, the comparisons summarized in this section should be interpreted as contextual rather than competitive. The numerical values reported in different studies are not used here to claim superiority or inferiority of DeepSnap because differences in data curation, class balance, endpoint definition, splitting strategy, and model-selection protocol can substantially affect reported performance.

### 8.2. Other Image-Based and Semi-Image-Based Molecular Representation Methods

DeepSnap is part of a broader family of molecular prediction approaches that use image-like inputs or computer-vision architectures. Fernandez et al. introduced Toxic Colors, which predicted Tox21 toxicity endpoints from 2D graphic images of compounds without relying on conventional molecular descriptors [12]. Chemception similarly used CNNs trained on 2D chemical structural drawings and demonstrated that image-based neural networks could learn chemically relevant features with minimal explicit chemical knowledge [13]. More recent large-scale image-based frameworks include ImageMol, a self-supervised molecular image pretraining strategy trained on millions of molecular images [14], and DEEPScreen, a drug–target interaction prediction system based on CNN learning from 2D structural compound representations [16]. Semianalogous approaches also exist, in which the input is not a rendered molecular picture but an image-like feature map derived from molecular descriptors or fingerprints. MolMap/MolMapNet maps molecular descriptors and fingerprint features into 2D feature maps that can be processed by CNNs [17].

These methods demonstrate that DeepSnap is not unique in applying computer-vision techniques to molecular prediction. The more precise distinction is representational. Toxic Colors, Chemception, ImageMol, and DEEPScreen primarily use 2D molecular depictions or structural images; MolMap/MolMapNet uses 2D feature maps derived from predefined descriptors and fingerprints; and DeepSnap uses conformer-derived 3D molecular images captured from multiple viewing angles. Within the DeepSnap framework, the utility of multiple viewing angles has already been examined. Previous DeepSnap studies showed that multiple molecular images captured from different angles outperformed single-image input and that the choice and combination of viewing angles can affect predictive performance [29,31]. Therefore, multiview image generation is not merely a conceptual feature of DeepSnap but an empirically motivated design element.

Graph neural networks and 3D-aware GNNs. GNNs represent the most prominent alternative paradigm in molecular property prediction. The foundational MPNN framework unified message passing over molecular graphs for property prediction [8], and subsequent developments such as D-MPNN and AttentiveFP have been extensively benchmarked on public datasets [9,10]. These models are primarily based on molecular topology, although additional descriptors or computed features may be incorporated depending on the application. Importantly, graph-based molecular learning is not limited to 2D topology. 3D-aware GNNs, exemplified by SchNet, incorporate geometric information, such as atomic coordinates or interatomic distances, into the learning architecture [11]. Thus, DeepSnap should not be framed as the only molecular learning approach capable of using 3D structural information. Rather, it differs from 3D-aware GNNs in representing prepared conformers as multiple 2D images for CNN input.

### 8.3. Descriptor-Based QSAR and DeepSnap Complementarity

Several DeepSnap studies included internal comparisons against conventional descriptor-based machine learning methods. In some Tox21 nuclear receptor and AhR studies, DeepSnap-DL showed higher ROC–AUC values than descriptor-based baselines under the reported conditions [29,31,32]. By contrast, for several ADME-related endpoints, descriptor-based models performed better than DeepSnap-DL alone, whereas ensemble or consensus models combining both representations improved performance or confidence [21,22,36]. Therefore, the most robust conclusion from the current literature is not the general superiority of DeepSnap but complementarity between molecular-image and descriptor-based representations.

### 8.4. Contextual Benchmarks: Tox21, CATMoS, BBBP, and Clearance

Previous versions of this manuscript repeated numerical contrasts with the Tox21 Data Challenge, CATMoS, MoleculeNet BBBP models, and clearance-pathway models. These comparisons have been abbreviated because the underlying protocols differ. CATMoS is an important reference point for acute oral toxicity prediction, but the CATMoS protocol and DeepSnap-based toxicity studies differ in model-development workflow and coverage [22,35]. Similarly, BBBP benchmarking in the MoleculeNet/DeepChem framework commonly emphasizes scaffold splitting, whereas the DeepSnap BBBP study used its own data-splitting and model-selection procedure [22,37,38]. Clearance-pathway comparisons provide useful context but were independently conducted rather than matched head-to-head evaluations [22,40,41]. A valid comparison would require re-evaluating DeepSnap and competing models on the same curated datasets with identical splits, preprocessing rules, hyperparameter-search budgets, and evaluation metrics.

Voxel-based 3D CNNs. A distinct class of 3D deep learning methods employs voxelized grids of protein–ligand complexes. AtomNet and the Gaussian atom density approach of Ragoza et al. demonstrated that 3D CNNs can learn binding features directly from structural data [46,47]. However, these methods address a different task domain—structure-based binding prediction requiring protein target structures—whereas DeepSnap performs ligand-based QSAR requiring only molecular structure. In addition, 3D convolutions scale cubically with spatial resolution, creating substantial computational costs that DeepSnap avoids through its 2D image projection strategy [24] (Table 2).

In conclusion, the current evidence supports the view that DeepSnap-derived image representations complement descriptor-based models, especially in ensemble or consensus settings. The inherent advantage of multiview DeepSnap images over single-image input should be distinguished from the lack of cross-method comparison with modern molecular representation models. No matched benchmark has yet established whether DeepSnap outperforms 2D molecular depiction methods, descriptor-derived image-like feature maps, topology-based GNNs, 3D-aware GNNs, or voxel-based approaches under common evaluation conditions. Therefore, future work should prioritize prospective matched benchmarking rather than further indirect comparison across publications.

## 9. Future Directions

The body of work reviewed in the preceding sections has established the DeepSnap-DL approach as a viable molecular-image-based QSAR methodology with demonstrated utility for toxicological endpoints and selected ADME/PK parameters. However, several limitations identified throughout this series of studies point toward concrete areas where further development could substantially strengthen the approach.

### 9.1. Matched Benchmarking and Reporting Standards

Benchmarking under matched conditions is considered the most urgent methodological need. Future studies should compare DeepSnap with descriptor-based QSAR, topology-based GNNs, 3D-aware GNNs, other image-based molecular prediction methods, and descriptor-derived feature-map methods using the same curated datasets with identical preprocessing, conformer-generation settings, data splits, hyperparameter-search budgets, and metrics. Such benchmarking studies should include repeated random splits, scaffold splits, independent external test sets, and the explicit reporting of class balance and applicability domain. Recent work further supports the need for such rigorous benchmarking. Deng et al. performed a large-scale evaluation involving 62,820 models and showed that dataset size, representation type, evaluation settings, and activity cliffs can substantially influence molecular property-prediction results [49]. Community resources such as the Therapeutics Data Commons provide standardized drug-discovery datasets, data splits, evaluation strategies, and leaderboards, whereas activity-cliff-focused benchmarks such as MoleculeACE emphasize the need to evaluate chemically difficult cases, not only average random-split performance [50,51].

### 9.2. Standardized and Robust Image-Generation Protocols

Future DeepSnap studies should develop standardized image-generation protocols to reduce endpoint-specific over-tuning and improve reproducibility. Important steps include the transparent reporting of image resolution, atom-color scheme, background color, molecular orientation, conformer-generation settings, viewing-angle combinations, and augmentation procedures. In addition, rendering parameters should be selected using training/validation data only, with the final performance evaluated on independent test sets that are not used during parameter selection. Sensitivity analyses should determine whether performance is stable across reasonable ranges of image-rendering parameters.

### 9.3. Conformer-Ensemble DeepSnap Models

A natural extension of DeepSnap is to move from single- or representative-conformer images to conformer-ensemble image sets. In such a framework, multiple low-energy conformers, force-field-generated conformers, or molecular-dynamics-derived snapshots could be rendered into multiview molecular images. Predictions could then be aggregated by simple averaging, probability ensembling, attention-based pooling, or multiple-instance learning. This strategy would allow DeepSnap to represent conformational heterogeneity while preserving its core image-based CNN architecture. This may be particularly important for flexible molecules and endpoints influenced by solution-state conformational equilibria or ligand–target binding adaptation. This direction should also be evaluated in the context of recent large-scale three-dimensional molecular representation learning. For example, Uni-Mol explicitly incorporates three-dimensional molecular information and was pretrained on large-scale molecular conformations and protein-pocket data [52]. Such coordinate-based three-dimensional pretraining frameworks provide useful reference points for assessing whether image-based conformer ensembles add information beyond existing three-dimensional molecular representation models.

### 9.4. Hybrid Image–Graph Models

Many GNNs used in ligand-based molecular property prediction use 2D molecular topology, whereas 3D-aware GNNs such as SchNet incorporate molecular geometry through atomic coordinates, interatomic distances, or related geometric features [11]. DeepSnap represents the same broad category of 3D structural information in a different form, i.e., conformer-derived multiview molecular images processed by CNNs. Therefore, future work should compare DeepSnap not only with conventional 2D GNNs but also with 3D-aware GNNs under matched datasets, conformer-generation procedures, data splits, and evaluation metrics. Beyond benchmarking, hybrid models could combine DeepSnap-derived image features or prediction probabilities with graph-derived representations from topology-based or 3D-aware GNNs. Whether such integration improves predictive performance, robustness, or interpretability remains to be confirmed. Recent geometry-aware and self-supervised graph methods provide appropriate modern comparators and potential feature sources for such hybrid systems. Examples include GEM, which incorporates topology and molecular geometry through a geometry-based GNN architecture and geometry-level pretraining; 3D Infomax, which uses three-dimensional pretraining to improve molecular GNN representations; and MolCLR, which applies contrastive self-supervised learning to molecular graphs using large unlabeled molecular datasets [53,54,55].

### 9.5. Systematic Interpretability Analysis

The interpretability of DeepSnap-DL models has been identified as a persistent limitation [2,24]. The S-COPHY study provided peer-reviewed Grad-CAM visualizations for a DeepSnap-type model [23], and in the AI-SHIPS project, ScoreCAM and Guided Grad-CAM were implemented for CYP inhibition models [43,44]. Future work should extend interpretability analyses beyond the currently documented CAM-family visualizations and apply the additional techniques discussed by Matsuzaka and Uesawa [2]—including LIME, SHAP, Anchor, and integrated gradients—to DeepSnap molecular images across a wider range of endpoints and CNN architectures. A key goal should be to move from pixel-space heat maps toward chemically interpretable atom-, bond-, or substructure-level explanations. Future interpretation studies should also include quantitative XAI evaluation and chemically grounded explanations. Recent molecular GNN studies have proposed XAI-specific benchmarks, concept-whitening-based self-interpretable QSAR models, and substructure-aware training objectives for improving explanation quality [56,57,58]. These methods would provide useful methodological references when moving DeepSnap explanations from image-space heat maps toward atom-, bond-, or substructure-level interpretations.

### 9.6. Expanding the Endpoint Repertoire and Multitask Learning

DeepSnap-DL has been applied mainly to nuclear receptor and stress response endpoints from the Tox21 library [19,20], rat clearance [21,36], and a limited set of additional drug screening endpoints [22]. Several ADME and toxicological endpoints of pharmaceutical relevance remain untested, including metabolic stability, plasma protein binding, hERG channel inhibition, hepatotoxicity, and genotoxicity. The S-COPHY model demonstrated that the molecular image concept could be adapted to chemical categorization tasks beyond toxicological activity [23]. In addition, the current DeepSnap-DL framework constructs independent models for each endpoint. Multitask learning architectures that predict multiple endpoints simultaneously could improve prediction efficiency and potentially benefit data-scarce endpoints through shared representation learning. Expansion to ADME endpoints should also be compared with recent public ADMET platforms and multitask frameworks. For example, ADMET-AI trains Chemprop-RDKit models on multiple TDC ADMET datasets and deploys multitask models covering regression and classification ADMET endpoints [59]. Such resources could serve as external benchmarks when extending DeepSnap to new ADME and toxicity endpoints [60,61,62,63].

## 10. Conclusions

DeepSnap is a descriptor-free QSAR approach that converts prepared 3D molecular conformers into image representations and feeds them into CNNs for biological activity and property prediction. Since its introduction in 2018, the method has been developed through a series of studies that optimized rendering parameters, CNN architectures, and combination strategies across toxicological and pharmacokinetic endpoints.

The DeepSnap research program has demonstrated that conformer-derived molecular images can be used as CNN inputs for QSAR modeling and that image-based predictions can provide information complementary to that provided by descriptor-based models, particularly in ensemble and consensus frameworks [21,22,36]. Previous DeepSnap studies also highlighted the value of multiview image generation over single-image input [29,31]. These findings show that DeepSnap provides promising complementary molecular representations and is not a fully benchmarked replacement for established descriptor-, graph-, or image-based approaches.

The high ROC–AUC values reported for endpoints such as CAR, PR antagonist prediction, BBBP, and hepatic clearance demonstrate strong discrimination within the datasets and validation protocols used in early DeepSnap studies. Nevertheless, high ROC–AUC values do not indicate that the predictions are deterministic, near-perfect, or universally generalizable. They may reflect endpoint-specific optimization of molecular-image generation, conformer preparation, viewing-angle selection, model hyperparameters, class distribution, endpoint definition, dataset composition, and training/testing split composition. Although several DeepSnap studies compared their results with independently published models or benchmark studies, most comparisons were indirect because of the differences in datasets, compound selections, splitting strategies, endpoint definitions, or evaluation metrics. Comprehensive matched external benchmarking against descriptor-based QSAR models, topology-based GNNs, 3D-aware GNNs, and other image-based molecular prediction methods remains incomplete [64,65,66,67,68,69].

Additional limitations include the reliance on static or representative-conformer-derived images, the dependence on nonphysical image-rendering parameters, limited systematic interpretability analysis, and the incomplete reporting of complementary evaluation metrics for imbalanced endpoints. Because prediction scores are not available for all published studies, this review did not recalculate PR-AUC, calibration, or confusion-matrix-based metrics. Future studies should complement ROC–AUC with PR-AUC values, balanced accuracy, MCC, sensitivity, specificity, confusion matrices, calibration analysis, repeated-split validation, scaffold-split validation, and independent external test sets [70,71,72].

Future priorities for DeepSnap development include standardized image-generation protocols, conformer-ensemble and molecular-dynamics-informed extensions, systematic explainable AI analyses [73], controlled benchmarking against 2D and 3D molecular representation models, automated hyperparameter optimization, and expansion to pharmacological and toxicological endpoints not yet examined. The consistent benefit of ensemble strategies suggests that the greatest practical impact of DeepSnap may lie not as a standalone method but as a complementary component within transparent, rigorously benchmarked, multirepresentation prediction systems.

## Figures and Tables

**Table 2:** Comparison of molecular property prediction approaches. Benchmark relation: indirect = same dataset or endpoint family but different split/protocol; none = no matched benchmark with DeepSnap under identical datasets, data splits, preprocessing procedures, and evaluation metrics. Abbreviations: 2D, two-dimensional; 3D, three-dimensional; DL, deep learning; DNN, deep neural network; DTI, drug–target interaction; ECFP, extended-connectivity fingerprint; Ens., ensemble; GNN, graph neural network; Interpr., interpretability; Med., medium; ML, machine learning; RF, Random Forest.

Family	Example	Representation	3D	Interpr.	Cost	Benchmark Relation
Desc. ML	RF/DNN + ECFP [48]	Descriptors/fingerprints	Partial	Med.	Low	Indirect: Tox21
Desc. DL	DeepTox [7]	ECFP + toxicophores	No	Low	Med.	Indirect: Challenge
Topo GNN	MPNN [8]	Molecular graph	No	Low	Med.	No matched DeepSnap benchmark
Topo GNN	D-MPNN [9]	Directed graph + RDKit	No	Low	Med.	Indirect: MoleculeNet
Attention GNN	AttentiveFP [10]	Graph + attention	No/opt.	Med.	Med.	No matched benchmark
3D-aware GNN	SchNet [11]	Atomistic graph + geometry	Yes	Low–Med.	Med.–High	No matched benchmark
2D Image	Toxic Colors [12]	2D graphic images	No	Low	Low	Indirect: Tox21
2D Image	Chemception [13]	2D structure drawings	No	Low	Low	Indirect: Tox21
2D Image	ImageMol [14]	2D images + pretraining	No	Med.	High	Indirect: BBBP overlap
2D Image	DEEPScreen [16]	2D structural images	No	Low–Med.	Med.	DTI task; no DeepSnap benchmark
Feature-map CNN	MolMapNet [17]	Descriptor/fingerprint maps	Partial	Med.	Med.	Semi-image; descriptor-derived
3D Voxel	AtomNet [46]	Voxelized complex	Yes	Low	High	Structure-based task
3D Voxel	Ragoza et al. [47]	Voxelized densities	Yes	Low	High	Structure-based task
DeepSnap	DeepSnap-DL [18,19]	Multiview 3D molecular images	Yes	Low–Med.	Med.	Reference method
DeepSnap Ens.	DeepSnap + ML [21,22]	Images + descriptors	Yes	Med.	Med.	Reference method

## Data Availability

No new data were created or analyzed in this study. Data sharing is not applicable to this article.
